# Supplementary material for: Content-rich biological network constructed by mining PubMed abstracts
Source: BMC Bioinformatics. 2004 Oct 8;5:147. doi: 10.1186/1471-2105-5-147 (PMC528731; doi:10.1186/1471-2105-5-147)
Supplement: Additional File 2 — The original results of the above study (non-essential files are deleted to keep the file size under the limit set by BMC bioinformatics). [file 1471-2105-5-147-S2.bz2 › chilibotAdditionalFile2/dip10/240ID9063449E787/html/MMP1_TIMP1.html]

 


 **MMP1** and **TIMP1** 
  
Found 2107 abstracts in PubMed, retrieved 10.  
 

 What does Google say? 
 PDF only 
| .edu only 

---

**Interactive relationship** (e.g. stimulation, inhibition, etc)

**Inhibitory relationship**- The minidegradome constituted of two matrix metalloproteinases MMP  [ **MMP1** ] , i.e. MMP  [ **MMP1** ]  2 and MT1 MMP  [ **MMP1** ] , associated with tissue inhibitor of metalloprotease 2 TIMP  [ **TIMP1** ]  2 and integrin alpha v beta 3 or CD 44, is mainly involved in such invasive program.  Ref: 12888258 Biomed Pharmacother, 2003
**Neutral relationship**

**Non-interactive relationship** (e.g. studied together, co-existance, homology, etc.)

- Low dose estrogen could decrease the ratio of MMP 1  [ **MMP1** ]  TIMP 1  [ **TIMP1** ]  and MMP  [ **MMP1** ]  3 TIMP 1  [ **TIMP1** ] , which could even be decreased by progestin.  Ref: 12887766 Zhonghua Yi Xue Za Zhi, 2003
- Our findings establish an unexpected, MMP  [ **MMP1** ]  independent mechanism for TIMP  [ **TIMP1** ]  2 inhibition of endothelial cell proliferation in vitro and reveal an important component of the antiangiogenic effect of TIMP2 in vivo.  Ref: 12887919 Cell, 2003
- OBJECTIVES The present study was carried out to clarify the role of matrix metalloproteinase 2 and 9 MMP  [ **MMP1** ]  2 and MMP  [ **MMP1** ]  9, tissue inhibitor of metalloproteinase 1  [ **TIMP1** ]  TIMP 1  [ **TIMP1** ]  and the extent of neovascularization in the clinicopathologic behavior of non Hodgkin s lymphomas.  Ref: 12890147 Eur J Haematol, 2003
- We demonstrate here that TIMP  [ **TIMP1** ]  2 abrogates angiogenic factor induced endothelial cell proliferation in vitro and angiogenesis in vivo independent of MMP  [ **MMP1** ]  inhibition.  Ref: 12887919 Cell, 2003
- Among the patients with strong grades 2 and 3 MMP  [ **MMP1** ]  9 staining positivity for TIMP 1  [ **TIMP1** ]  indicated a trend toward a more favorable prognosis.  Ref: 12890147 Eur J Haematol, 2003
- Reverse transcription polymerase chain reaction were used to detect the mRNA expression of MMP 1  [ **MMP1** ] , MMP  [ **MMP1** ]  3, TIMP 1  [ **TIMP1** ]  and IL 1beta in the OA synovia to determine the effect of estrogen and progestin.  Ref: 12887766 Zhonghua Yi Xue Za Zhi, 2003
- Platelet derived growth factor BB was generally ineffective in modulating MMP  [ **MMP1** ]  and TIMP  [ **TIMP1** ]  levels.  Ref: 12882798 Invest Ophthalmol Vis Sci, 2003
- 0.001, and the MMP  [ **MMP1** ]  inhibitor TIMP  [ **TIMP1** ]  2 P = 0.025.  Ref: 12893184 Gynecol Oncol, 2003
- Our results showed a significant decrease of collagenases MMP  [ **MMP1** ]  8 and MMP  [ **MMP1** ]  13 with an increase of TIMP 1  [ **TIMP1** ]  and transforming growth factor beta.  Ref: 12882763 Am J Physiol Lung Cell Mol PhysiolAm J Physiol Lung Cell Mol Physiol, 2003
- OBJECTIVE To observe the effect of different dose of estrogen or estrogen plus progestin on the mRNA expression of MMP 1  [ **MMP1** ] , MMP  [ **MMP1** ]  3, TIMP 1  [ **TIMP1** ]  and IL 1beta in synovia of female New Zealand White rabbit OA model.  Ref: 12887766 Zhonghua Yi Xue Za Zhi, 2003
- The purpose of this study was to characterize effects of cytokines, a phorbol ester, and prostanoids on the expression of MMP  [ **MMP1** ] , 3, and 9 and tissue inhibitors of metalloproteinases TIMP 1  [ **TIMP1** ]  and 2 in cultured human TM cells.  Ref: 12882798 Invest Ophthalmol Vis Sci, 2003
- The expression of collagenases MMP  [ **MMP1** ]  and 13 and tissue inhibitor of metalloproteinases  [ **TIMP1** ]  TIMP 1  [ **TIMP1** ]  was altered in OA cartilage, but no difference was detected between lesion and distal sites in the same jointi.e. no correlation was found between senescent cells and proteinase inhibitor expression.  Ref: 12882354 Aging Cell, 2002
- However, we were unable to demonstrate any differences in the constitutive productions and secretions of MMP  [ **MMP1** ]  2, MT1 MMP  [ **MMP1** ] , and TIMP  [ **TIMP1** ]  2, among the mock treated, Ad lacZ transduced, and Ad p16 transduced cells.  Ref: 12883667 Oncol RepOncol Rep, 2002
- However, the mRNAs coding for urokinase type PA uPA, membrane type 1 MMP  [ **MMP1** ]  MT1 MMP  [ **MMP1** ] , and tissue inhibitor of metalloproteinases  [ **TIMP1** ]  type 3 TIMP  [ **TIMP1** ]  3 were constantly expressed in the mouse CL throughout its whole life span.  Ref: 12890575 Mol Cell Endocrinol, 2003
- METHODS Paraffin embedded histologic sections from 57 patients with aggressive non Hodgkin s lymphomas were stained with MMP  [ **MMP1** ]  2, MMP  [ **MMP1** ]  9, TIMP 1  [ **TIMP1** ] , and factor VIII antibodies to correlate the expression of these markers to the clinical disease characteristics.  Ref: 12890147 Eur J Haematol, 2003
- In this study we examined the sequential expression of several matrix metalloproteinases MMP  [ **MMP1** ] , tissue inhibitors of metalloproteinases TIMP  [ **TIMP1** ] , and growth factors as well as the presence of apoptosis in a model of pulmonary fibrosis induced in rats with paraquat and hyperoxia.  Ref: 12882763 Am J Physiol Lung Cell Mol PhysiolAm J Physiol Lung Cell Mol Physiol, 2003
- Gelatinases MMP  [ **MMP1** ]  2 and MMP  [ **MMP1** ]  9, TIMP 1  [ **TIMP1** ]  expression and the extent of neovascularization in aggressive non Hodgkin s lymphomas.  Ref: 12890147 Eur J Haematol, 2003
- The invasive potential of Ad p16 transduced cells was evaluated using a Matrigel invasion assay, and trimolecular complex MMP  [ **MMP1** ]  2 MT1 MMP  [ **MMP1** ]  TIMP  [ **TIMP1** ]  2 synthesis was proven by zymography and Western blotting.  Ref: 12883667 Oncol RepOncol Rep, 2003
